# Artifact-free and high-temporal-resolution in vivo opto-electrophysiology with microLED optoelectrodes

**DOI:** 10.1038/s41467-020-15769-w

**Published:** 2020-04-28

**Authors:** Kanghwan Kim, Mihály Vöröslakos, John P. Seymour, Kensall D. Wise, György Buzsáki, Euisik Yoon

**Affiliations:** 1 Department of Electrical Engineering and Computer Science, University of Michigan, Ann Arbor, MI 48109 USA; 2 Neuroscience Institute, Langone Medical Center, New York University, New York, NY 10016 USA; 3 Department of Biomedical Engineering, University of Michigan, Ann Arbor, MI 48109 USA; 4 Center for Nanomedicine, Institute for Basic Science (IBS) and Graduate Program of Nano Biomedical Engineering (Nano BME), Yonsei University, Seoul, 03722 Korea

**Keywords:** Biosensors, Neural circuits, Biomedical engineering, Electrical and electronic engineering

## Abstract

The combination of in vivo extracellular recording and genetic-engineering-assisted optical stimulation is a powerful tool for the study of neuronal circuits. Precise analysis of complex neural circuits requires high-density integration of multiple cellular-size light sources and recording electrodes. However, high-density integration inevitably introduces stimulation artifact. We present minimal-stimulation-artifact (miniSTAR) μLED optoelectrodes that enable effective elimination of stimulation artifact. A multi-metal-layer structure with a shielding layer effectively suppresses capacitive coupling of stimulation signals. A heavily boron-doped silicon substrate silences the photovoltaic effect induced from LED illumination. With transient stimulation pulse shaping, we reduced stimulation artifact on miniSTAR μLED optoelectrodes to below 50 μV_pp_, much smaller than a typical spike detection threshold, at optical stimulation of >50 mW mm^–2^ irradiance. We demonstrated high-temporal resolution (<1 ms) opto-electrophysiology without any artifact-induced signal quality degradation during in vivo experiments. MiniSTAR μLED optoelectrodes will facilitate functional mapping of local circuits and discoveries in the brain.

## Abstract (web summary)

Artifact-free opto-electrophysiology is key for precise modulation and monitoring of individual neurons at high spatio-temporal resolution. The authors present a method for eliminating stimulation artifacts in high-density micro-LED optoelectrodes for accurate functional mapping of local circuits.

## Introduction

A brain is made up of densely populated neurons. Analysis of neuronal communication requires simultaneous high-resolution recording and neuron-specific perturbation of circuit components under controlled conditions. The combination of genetic-engineering-assisted optical stimulation and massively parallel electrical recording of neuronal activities (opto-electrophysiology) is a promising tool for studying neuronal circuits in behaving animals^1^. A number of devices^2–12^ have been introduced for the past few years for in vivo opto-electrophysiology. For high-resolution in vivo opto-electrophysiology, a micromachined silicon multi-electrode-array structure also known as the Michigan Probe^13,14^ has been widely utilized^6–11^. Desirable material properties, low cost, and ease of micromachining had made silicon, or more specifically single-crystalline silicon, the best material choice for neural interfaces over other rigid materials such as metal, quarts, soda lime glass, and sapphire^14^. The minimal cross-sectional area (typically < 70 μm × 30 μm) of the electrode arrays the Michigan Probe platform enables makes the silicon electrode arrays the primary choice for both acute and chronic in vivo extracellular electrophysiology applications^15^. The silicon optoelectrodes, in addition, take advantage of the planar profile of the platform and accommodate multiple light sources in the vicinity of high-density recording electrode arrays. This compact configuration provides the capability to electrically record the activity of sets of neurons at high-spatial resolution while optically stimulating a portion of the recorded neurons.

An undesirable feature of many of these devices is the stimulation artifact. With its magnitude often an order of magnitude larger than those of underlying neuronal signals, the stimulation artifact may mask neuronal signals and prevent the temporally precise recording of neuronal responses^16,17^. In order to enable precise detection of neuronal activities, the magnitude of the stimulation artifact should be reduced to lower than a threshold voltage level for neuronal activity detection. Typically, the threshold level is set as a few integer multiples (often 5×) of the root-mean-square value of background noise^18,19^. To keep the artifact magnitude lower than the threshold level, optical stimulation had been limited to slowly changing, low-frequency pulses, such as slow (<10 Hz) sine waves^11^ or trapezoidal pulses with a long (>10 ms) rise time^20^. These slowly changing optical stimulation protocols, however, are not suitable for many neuroscience experiments in which high-speed neuromodulation is required, such as those in closed-loop experimental setups^21^. An ideal optoelectrode should, therefore, provide optical stimulation with a temporal resolution higher than the duration of the neuronal activities while keeping the stimulation artifact magnitude lower than a spike detection threshold.

We present minimal-stimulation-artifact (miniSTAR) µLED optoelectrode and report the engineering schemes that enabled artifact-free optical stimulation. We extensively characterized various forms of optical-stimulation-induced artifacts, including photoelectrochemical effects (PEC)^22–27^, electromagnetic interference (EMI)^6,28,29^, and photovoltaic effects (PV)^30,31^. MiniSTAR optoelectrodes utilize monolithically integrated neuron-sized LEDs^11^ for high-spatial resolution optical stimulation of target neurons. The multi-metal-layer structure on the miniSTAR optoelectrode suppresses EMI-induced stimulation artifact, and the heavily boron-doped silicon substrate eliminates PV-induced artifact. Additionally, transient pulse shaping control reduces the magnitude of residual stimulation artifact on all recording channels to <50 μV peak-to-peak (μVpp) without compromising the temporal resolution of optical stimulation. With an in vivo experiment using a miniSTAR optoelectrode implanted in a mouse brain, we demonstrate the absence of distortion in the recorded neuronal signals during precise in situ optical stimulation.

## Results

### MiniSTAR μLED optoelectrode fabrication

**Fig. 1:**
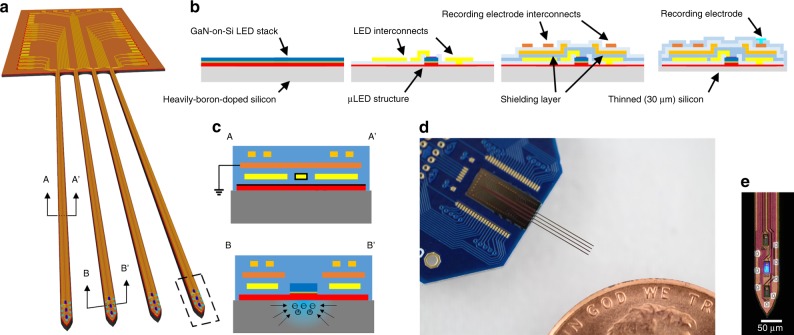
MiniSTAR optoelectrode. **a** Schematic illustration of a miniSTAR μLED optoelectrode. **b** Simplified miniSTAR optoelectrode fabrication process. An illustrative cross-section containing only one LED and one recording electrode is shown. **c** Cross-sectional schematic diagrams of a shank of a miniSTAR optoelectrode, showing sources of stimulation artifact (EMI and PV effect for top and bottom, respectively) and methods for reduction of stimulation artifact. **d** Photograph of a miniSTAR optoelectrode mounted on a PCB. **e** Microphotograph of a tip of a miniSTAR optoelectrode, on which eight recording electrodes, three LEDs, LED interconnects, shielding layer, and recording electrode interconnects are shown.

We fabricated miniSTAR μLED optoelectrodes (Fig. 1a) using microfabrication techniques adapted from those used for the fabrication of the family of Michigan optoelectrodes including one-metal-layer μLED optoelectrodes^11^. Figure 1b describes the simplified device fabrication flow. MiniSTAR optoelectrodes were fabricated using gallium-nitride-on-silicon (GaN-on-Si), gallium nitride/indium gallium nitride multi-quantum-well (GaN/InGaN MQW) LED wafers with heavily boron-doped silicon (p^+^-Si, *N*_A_ ≈ 1 × 10^20^ cm^–3^) substrates. In order to reduce EMI-induced stimulation artifact, metal traces for LED drive signals (LED interconnects) and those for recorded neuronal signals (recording electrode interconnects) were placed in two different metal layers separated from each other by a ground-connected shielding layer (Fig. 1c, top), forming a multi-metal-layer structure. A heavily boron-doped substrate was chosen to suppress diffusion of optically generated electron-hole pairs and, as a result, to reduce PV-induced stimulation artifact (Fig. 1c, bottom). First, LED mesa structures were formed on the GaN/InGaN MQW layer, and the LED interconnects were defined on the first metal layer. After passivating the surface of the LEDs, the EMI shielding layer was defined on the second metal layer and the recording electrode interconnects were defined on the third metal layer. Neural signal recording electrodes were then formed by depositing electrode material (iridium) on top. Finally, the entire wafer was thinned down to 30 μm and the miniSTAR optoelectrodes were released from the silicon wafer. Released miniSTAR optoelectrodes were assembled on printed circuit boards (PCBs) that provide connections to a neuronal signal recording IC and an LED driver (Fig. 1d). Figure 1e shows a microphotograph of a tip of the fabricated miniSTAR optoelectrode. The dimensions of the exposed surface area of each μLED and recording electrodes are 10 μm × 15 μm and 11 μm × 13 μm (*W* × *L*), respectively.

After fabricating the miniSTAR optoelectrodes, we characterized the performance of the LEDs and recording electrodes and confirmed that they are suitable for in vivo opto-electrophysiology. Optical power equivalent to greater than 1 mW mm^–2^ of irradiance is considered a threshold for activation of channelrhodopsin-2 (ChR2)^9,11^. LEDs on miniSTAR optoelectrodes generated a radiant flux of 150 nW, equivalent to an irradiance of 1 mW mm^–2^ at the surface when a voltage of 2.86 ± 0.02 V (mean ± SD, *n* = 22) was applied across their terminals. The LEDs were capable of generating 50 mW mm^–2^ at the surface (7.5-μW radiant flux) at 3.46 ± 0.10 V, which is more than sufficient for activation of ChR2-expressing cells further away from the LED surface. We confirmed that the effect of substrate doping density on the electrical and optical characteristics of the fabricated LEDs is not as significant as the die-to-die variation in a wafer (Supplementary Fig. 1). The impedance magnitude and phase of the recording electrodes were measured as 1.15 ± 0.07 MΩ and –68.33 ± 5.11 ° at 1 kHz (*n* = 54, mean ± SD), respectively, acceptable for high-quality in vivo extracellular recordings^32^.

### Reduction of EMI-induced artifact

EMI is inevitable in a system where a source of a high-voltage, fast-changing signal is located in close proximity to a signal-carrying trace connected to a high-impedance load. Previous μLED optoelectrodes^11^ contained only one metal layer on which all the interconnects that carry optical stimulation signals, as well as those carrying recorded neural signals, were densely integrated. Therefore, mutual capacitances between the traces of two signal types were high, and, in turn, the recording interconnects were highly susceptible to EMI from LED drive signals. Moreover, the n-GaN layer that forms the common cathode of all the μLEDs on the optoelectrode was directly underneath the interconnects and acted as another significant source of EMI. FEM simulations of electrostatic potential distribution in the one-metal-layer μLED optoelectrode (Supplementary Note 2 and Supplementary Fig. 2c) showed significant voltage coupling from LED interconnects (–48.96 dB), as well as from the n-GaN layer (–0.06 dB).

We observed significant suppression of EMI-induced stimulation artifact with the integration of a shielding layer. We implemented the triple-metal-layer structure on μLED optoelectrode and dedicated a layer between the stimulation and recording interconnects as a shielding layer (Supplementary Fig. 2d). Triple-metal-layer (shielded) μLED optoelectrodes were fabricated on the same GaN-on-Si LED wafer on which one-metal-layer μLED optoelectrodes were fabricated, which had a lightly boron-doped silicon substrate (*N*_A_ ≈ 5 × 10^16^ cm^–3^). We compared stimulation artifacts between one-metal-layer μLED optoelectrodes and shielded μLED optoelectrodes while turning on and off μLEDs in vitro.

**Fig. 2:**
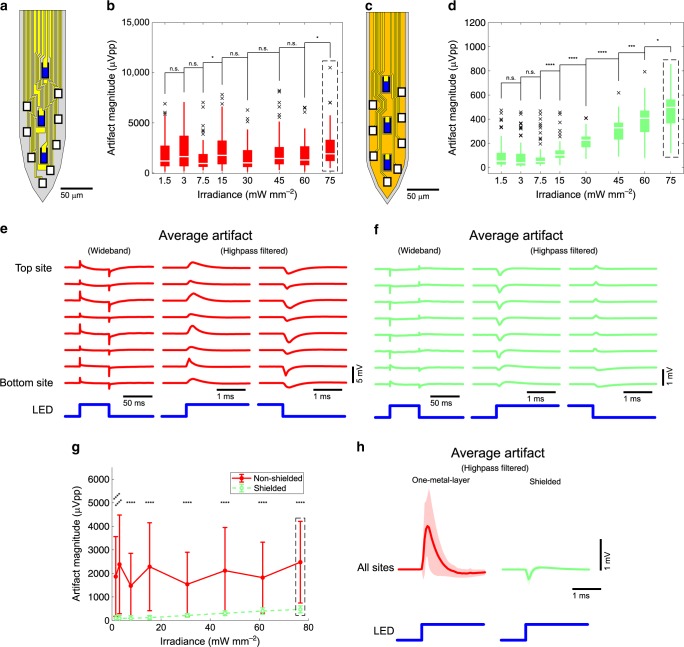
Reduced EMI-induced artifact. **a** Schematic illustration of the tip of one-metal-layer (non-shielded) μLED optoelectrode. Blue rectangles indicate LEDs, white rectangles the recording electrodes, and yellow polygons interconnects. **b** The peak-to-peak magnitude of highpass filtered stimulation artifact recorded on non-shielded μLED optoelectrodes. Data from channels corresponding to all electrodes on the shank on which an LED was turned on are plotted. Boxes indicate interquartile ranges, white lines medians, whiskers non-outlier extreme values, and black x marks outliers. **c** Schematic illustration of the tip of shielded μLED optoelectrodes. The color scheme is identical to that of part **a**, except for additional color, gold, to indicate the shielding layer. **d** The peak-to-peak magnitude of highpass filtered stimulation artifact recorded on shielded μLED optoelectrodes. **e** Mean waveforms of stimulation artifact recorded on non-shielded μLED optoelectrodes, from channels that correspond to electrodes on different locations on the tips. LED drive signal with resulting LED surface irradiance of 75 mW mm^–2^ was used. **f** Mean waveforms of stimulation artifact recorded on shielded μLED optoelectrodes. LED drive signal with resulting LED surface irradiance of 75 mW mm^–2^ was used. **g** Comparison of mean peak-to-peak magnitudes of highpass filtered stimulation artifact recorded on the shielded (green) and the non-shielded (red) μLED optoelectrodes. Error bars indicate one standard deviation. **h** Mean highpass filtered waveforms whose mean peak-to-peak magnitudes are shown in part **g**, inside the rectangle with black dashed lines. Shaded regions show one standard deviation away from the mean. Mean (±SD) peak-to-peak magnitudes are 2477.8 (±1733.83) μVpp for non-shielded μLED optoelectrodes (*n* = 75) and 474.6 (±146.26) μVpp for shielded μLED optoelectrodes (*n* = 67). The results of statistical tests are provided in Supplementary Table 2.

Figure 2 shows the magnitude of the transient stimulation artifact (peak-to-peak) and the wideband and highpass filtered waveforms of the artifacts resulting from optical stimulation. One-metal-layer μLED optoelectrodes showed a high magnitude (>1 mVpp) in most recording sites regardless of the amount of optical power generated from the LEDs (Fig. 2b). On the other hand, shielded optoelectrodes showed significantly smaller stimulation artifact (100–400 μVpp), whose magnitude gradually increases at larger irradiance (Fig. 2d). The shape and the phase of the wideband stimulation artifacts on one-metal-layer LED optoelectrodes (Fig. 2e, left) suggest a strong contribution of EMI. Once highpass filtered, the artifact generated an “inverted v” shaped transient at the onset of the optical stimulation (or, in other words, at the rising edge of the stimulation signal) and a “v” shaped transient at the end of the optical stimulation (or at the falling edge of the stimulation signal). The weak dependence of stimulation artifact magnitude on the optical power suggests that voltage coupling from the n-GaN substrate, whose voltage does not greatly change as a function of the LED signal voltage, might contribute to the EMI-induced stimulation artifact greater than those from LED interconnects do.

Great reduction in the stimulation artifact magnitude was consistent with the expectation from the FEM simulations (Supplementary Fig. 2d). A significant reduction of the stimulation artifact on shielded optoelectrodes was observed at all irradiance under test (Fig. 2g). At 75-mW mm^–2^ irradiance (radiant flux of 11.5 μW), we achieved 5.2-fold reduction in stimulation artifact (from 2477.75 ± 1733.83 to 474.59 ± 146.26 μVpp, *n* = 75 and 67, respectively).

### Elimination of PV-induced artifact

Although the EMI-induced artifact was greatly suppressed with the introduction of the shielding layer, the magnitude of the residual artifact was still high and should be further reduced below that of typical neuronal spikes (~100 μVpp). Interestingly, we noticed that the polarities of the stimulation artifact at the onset and the end of an optical stimulation (in other words, the rising and the falling edges of a LED drive pulse) became inverted on the shielded μLED optoelectrodes. As can be seen in Fig. 2h (left), the transient artifact on one-metal-layer μLED optoelectrodes has an “inverted-v” (or “^”) shaped waveform at the onset of the optical stimulation. However, on the shielded μLED optoelectrodes (Fig. 2h, right), the polarity of the transient artifact was inverted, making a “v”-shaped waveform. The shape of the transients at the end of the optical stimulation became inverted as well. Inversion of the polarity of the transient artifact suggested that the residual artifact could have resulted from a different source other than EMI.

We hypothesized that the source of the v-shaped stimulation artifacts is photovoltaic (PV) effects in the silicon substrate and confirmed our hypothesis with a few experiments. First, we observed the waveform of signals recorded on electrodes while exposing the μLED optoelectrodes to external optical illumination. Using a focused beam at a wavelength similar to that of the light generated from μLEDs (*λ*_peak_ ≅ 470 nm), we illuminated tips of the shielded μLED optoelectrodes. The shape of the induced voltage signal was identical to that of the stimulation artifact observed on the optoelectrodes (Supplementary Fig. 3). The identical shape suggested that the artifact is truly optically induced, not resulting from EMI. We repeated the experiment using electrode arrays fabricated on non-silicon substrates:GaN-on-sapphire wafer and soda lime glass. We did not observe any v-shaped stimulation artifacts on electrodes on both substrates (Supplementary Fig. 4), verifying that the artifact is due to neither photoelectrochemical (PEC) effects on the electrodes nor PV-induced artifact on the GaN layer. With the exclusion of PEC effects and PV effect from the GaN layer, the only remaining source of potential light-induced artifact is the PV effects from the silicon substrate.

A few experimental studies in the past reported that light-induced noise on silicon electrode arrays can be reduced with the use of heavily doped substrate^33,34^. Heavy doping of semiconductor greatly reduces carrier lifetimes^35,36^ and diffusion lengths of free carriers, which supposedly contributes to the amount of dipole-induced voltage^33^. Therefore, PV-induced stimulation artifacts should be suppressed with heavy doping of the silicon substrate. We conducted FEM simulations of optically induced voltage generation in silicon substrates and verified that the voltage is reduced with heavy substrate doping. We built a model of the silicon substrate and calculated the optically induced voltage generation while changing doping concentrations (Supplementary Methods). The results suggest that a highly boron-doped silicon substrate can greatly reduce the magnitude of optically induced voltage and as a result suppress PV-induced artifact (Supplementary Note 3 and Supplementary Fig. 5).

**Fig. 3:**
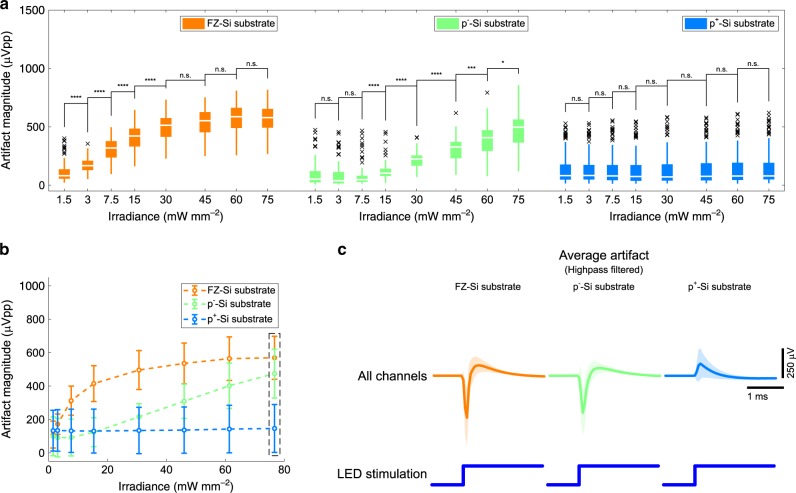
Eliminated PV-induced artifact. **a** Peak-to-peak magnitude of highpass filtered stimulation artifact recorded on shielded μLED optoelectrodes with different substrate doping densities. Data from channels corresponding to all electrodes on the shank on which an LED was turned on are plotted. Boxes indicate interquartile ranges, white lines medians, whiskers non-outlier extreme values, and black x marks outliers. **b** Comparison of the mean peak-to-peak magnitude of highpass filtered stimulation artifact whose distribution is shown in part **a**. Circles indicate the mean, and the error bars indicate one standard deviation. **c** Mean highpass filtered waveforms whose mean peak-to-peak magnitudes are shown in part **b**, inside the rectangle with black dashed lines. Shaded regions show one standard deviation away from the mean. The mean (±SD) peak-to-peak magnitudes are 569.33 (±129.00), 474.59 (±146.26), and 146.05 (±143.40) μVpp for devices with FZ-Si substrate (*n* = 124), p^−^-Si substrate (*n* = 67), and p^+^-Si substrate (*n* = 151), respectively. A detailed description of the samples, statistical tests used, and the results of statistical tests are provided in Supplementary Table 2.

In order to verify the effect of doping density on the magnitude of PV-induced stimulation artifact, we fabricated three groups of shielded μLED optoelectrodes using GaN-on-Si GaN/InGaN MQW LED wafers with different silicon substrates: float-zone grown silicon substrate (FZ-Si, *N*_A_ ≈ 4 × 10^12^ cm^–3^), lightly boron-doped silicon substrate (p^−^-Si, *N*_A_ ≈ 5 × 10^16^ cm^–3^), and heavily boron-doped silicon substrate (p^+^-Si, *N*_A_ ≈ 1 × 10^20^ cm^–3^). Figure 3a shows that the magnitude of stimulation artifact measured on the optoelectrodes fabricated using wafers with FZ-Si and p^−^-Si substrates increases as a function of irradiance (FZ-Si: 109.59 ± 80.61 μVpp at 1.5 mW mm^–2^ increasing to 569.33 ± 129.00 μVpp at 75 mW mm^–2^, p^−^-Si: 99.25 ± 116.01 μVpp at 1.5 mW mm^–20^ increasing to 474.59 ± 146.26 μVpp at 75 mW mm^–2^, mean ± SD). On the other hand, the stimulation artifact magnitude on devices with p^+^-Si substrate did not show any significant change (133.04 ± 121.99 μVpp at 1.5 mW mm^–2^ to 146.05 ± 143.4 μVpp at 75 mW mm^–2^, mean ± SD). The magnitude of stimulation artifact as a function of irradiance and substrate doping density (Fig. 3b) was similar to that expected from FEM simulation (Supplementary Fig. 5d). Figure 3c shows the waveforms of stimulation artifact measured on the optoelectrodes of each group. It can be seen that even with high-intensity illumination (11.5 μW, or 75 mW mm^–2^), the mean magnitude of stimulation artifact was below 200 μVpp, suggesting that the PV-induced stimulation artifact was effectively reduced by the use of heavily boron-doped silicon substrate.

**Fig. 4:**
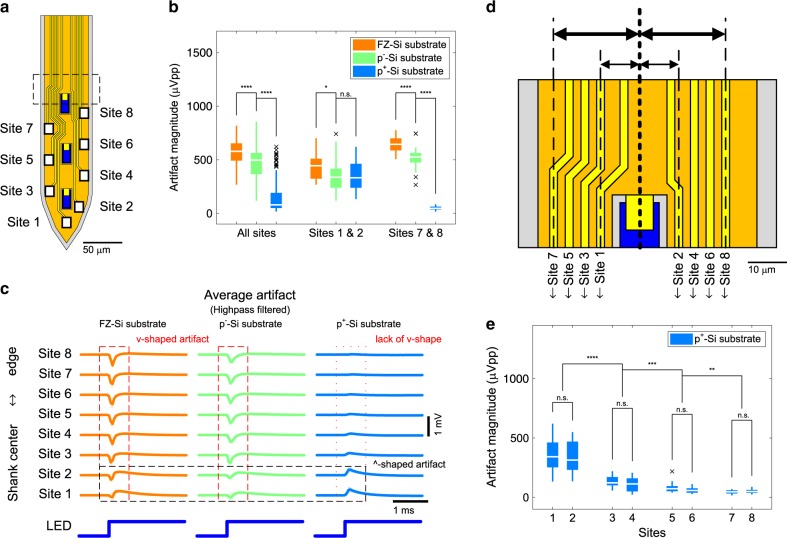
Location dependence of residual artifact. **a** Schematic illustration of the tip of shielded μLED optoelectrode. **b** The peak-to-peak magnitude of highpass filtered stimulation artifact recorded on shielded μLED optoelectrodes with different substrate doping densities. Data from channels corresponding to electrodes on the shank on which an LED was turned on are plotted. LED drive signal with resulting LED surface irradiance of 75 mW mm^–2^ was used. Boxes indicate interquartile ranges, white lines medians, whiskers non-outlier extreme values, and black x marks outliers. **c** Mean waveforms of stimulation artifact recorded on the shielded μLED optoelectrodes with different substrate doping densities, from channels that correspond to electrodes on different locations on the tips. Shaded regions show one standard deviation away from the mean. LED drive signal with resulting LED surface irradiance of 75 mW mm^–2^ was used. **d** Magnified view of the region inside the rectangle with the black dashed lines on part **a**. The distances between the center of the interconnects and the center of an LED are shown. **e** Peak-to-peak magnitudes of highpass filtered stimulation artifact recorded from different channels on shielded μLED optoelectrodes with heavily boron-doped silicon substrate (miniSTAR optoelectrodes). LED drive signal with resulting LED surface irradiance of 75 mW mm^–2^ was used. A detailed description of the samples, statistical tests used, and the results of statistical tests are provided in Supplementary Table 2.

We confirmed the elimination of the PV-induced stimulation artifact by inspecting the shape and the magnitude of stimulation artifact waveforms recorded from electrodes at different locations on μLED optoelectrodes (Fig. 4). Figure 4b shows the magnitude of stimulation artifact recorded from channels that correspond to the electrodes marked in Fig. 4a. The artifact waveform recorded from each channel is presented in Fig. 4c. It is interesting to note that, while the v-shaped waveform in stimulation artifact was observed in the recordings from optoelectrodes with FZ-Si and p^−^-Si substrates, we no longer observed the v-shape in those from the optoelectrodes with p^+^-Si substrates. The absence of the characteristic v-shaped waveform confirms that the PV-induced stimulation artifact has been eliminated on the optoelectrodes with p^+^silicon substrate.

### Suppression of residual EMI-induced artifact

Considering the great reduction of both EMI- and PV-induced stimulation artifact, we refer to the shielded μLED optoelectrodes fabricated using an LED wafer with p^+^ silicon substrate as minimal-stimulation-artifact (miniSTAR) μLED optoelectrodes. We quantified the amount of reduction in stimulation artifact from the implementation of shielding layers and the replacement of substrate with highly boron-doped silicon in miniSTAR optoelectrodes (Supplementary Fig. 6). The magnitude of artifact was reduced by a factor of 5.2 in average only from the use of the shielding layer (from 2477.75 ± 1733.83 to 474.59 ± 146.26 μVpp, at 11.5 μW, mean ± SD), and by a factor of 17 in average from both shielding and substrate replacement combined (to 146.05 ± 143.40 μVpp, at 11.5 μW, mean ± SD). However, the magnitude of stimulation artifact in a couple of recording sites (sites 1 and 2) was still high, as large as 200–300 μVpp, while those on some other sites (sites 7 and 8) were <50 μVpp (Fig. 4e).

Location dependence of the residual stimulation artifact revealed that the residual artifact is due to EMI resulting from imperfections in the shielding layer. The shieling layer on the miniSTAR optoelectrode contains openings (or optical windows) on top of μLEDs for illumination. However, the optical windows allow the electric field generated from the LEDs to exit the shielding layer and make the interconnects susceptible to EMI. Once the PV-induced artifact was removed in miniSTAR optoelectrodes, we observed the emergence of ^-shaped waveforms (Fig. 4c), which is especially pronounced on sites 1 and 2. The magnitude of ^-shaped waveform is inversely proportional to the distance between the interconnect for each site and the optical window on the shielding layer (Fig. 4c–e). The polarity and the distance dependence of stimulation artifact waveforms suggest that this residual artifact is at least partially due to EMI originating from the LEDs that are exposed through optical windows on the shielding layer.

**Fig. 5:**
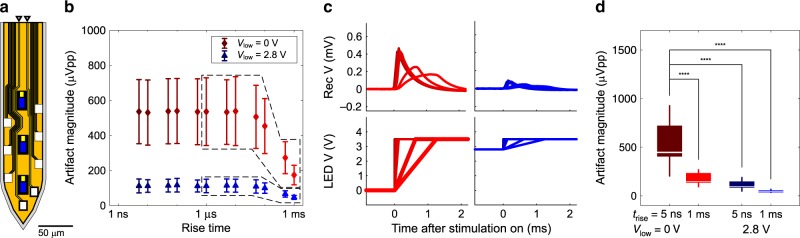
Effect of transient pulse shaping on residual artifact. **a** Schematic illustration of the tip of a miniSTAR μLED optoelectrode. Locations of the electrodes and the interconnects from which the signals were recorded are indicated with rectangles with bold black lines and black arrowheads, respectively. **b** Mean peak-to-peak magnitude of highpass filtered stimulation artifact recorded from the channels indicated in part **a** for two different low-level voltages (*V*_low_ = 0 V and *V*_low_ = 2.8 V). A high-level voltage of 3.5 V was used. *x* coordinates indicate the 10–90% rise time of the pulse, symbols (circle and triangle) indicate the mean, and error bars indicate one standard deviation (*n* = 35). **c** Mean waveforms of recorded stimulation artifact, whose mean peak-to-peak magnitudes are shown inside the polygon with dashed lines in part **b**, and their input voltage signals. Stimulation artifact resulting from an input voltage signal is indicated with the same color. **d** Peak-to-peak magnitudes of highpass filtered stimulation artifact for a few selected conditions whose means are shown in part **b**. Boxes indicate interquartile ranges, white lines medians, and whiskers extreme values. Mean (±SD) peak-to-peak magnitudes are 535.80 (±182.94), 173.99 (±55.76), 111.92 (±39.55), and 46.53 (±11.33), from left to right. A detailed description of the samples, statistical tests used, and the results of statistical tests are provided in Supplementary Table 2.

Additional suppression of residual artifact was achieved by transient pulse shaping of LED drive signals. We modified the slew rate of voltage pulses by changing the rise and fall times of the pulses. With a sufficiently long rise time (*t*_rise_ > (2π*F*_s_)^–1^), the magnitude of higher-order harmonics of the coupled signal that contributes to the artifact ((π*t*_rise_)^–1^ <  *f*  < *F*_s_/2) is reduced by an additional –20 dB per decade (Supplementary Fig. 7). Figure 5 shows the peak-to-peak magnitude and waveforms of stimulation artifact recorded from the channels corresponding to the bottom two electrodes (sites 1 and 2) on the tip of miniSTAR optoelectrodes, which show the worst residual EMI-induced artifact. We observed a significant reduction in stimulation artifact as we increased the rise time to longer than 100 μs. At 50 mW mm^–2^ irradiance, the artifact magnitude was reduced to below 200 μVpp (173.99 ± 55.76 μVpp, mean ± SD, for 1-ms rise time). In order to further reduce the slew rate of the voltage driving signal, we adjusted the low-level (or off-state) voltage in the stimulation pulse signals. We increased the low-level voltage of the signal provided to the LED anode to 2.8 V, just below the lowest turn-on voltage of LEDs. The LED cathode voltage was kept at 0 V. The voltage required for irradiance of 50 mW mm^–2^ (radiant flux of 7.5 μW) is ~3.5 V. By adjusting the low-level voltage from 0 V to 2.8 V, we reduced the voltage swing from 3.5 V to 0.7 V and the slew rate by a factor of 5. We confirmed that the artifact magnitude can be reduced to 111.92 μVpp (SD = 55.76 μVpp) even without adjusting the rise time (Fig. 5b, *V*_low_ = 2.8 V, blue). With a 1-ms rise time and 2.8-V low-level voltage, the mean artifact magnitude was reduced to 46.53 μVpp (SD = 11.33 μVpp). In typical in vivoextracellular measurements, 100 μVpp is used as a spike detection threshold due to biological and environmental noise. Therefore, stimulation artifact with less than 50-μVpp magnitude can be considered nearly artifact-free.

### Stimulation-artifact-free in vivo opto-electrophysiology

**Fig. 6:**
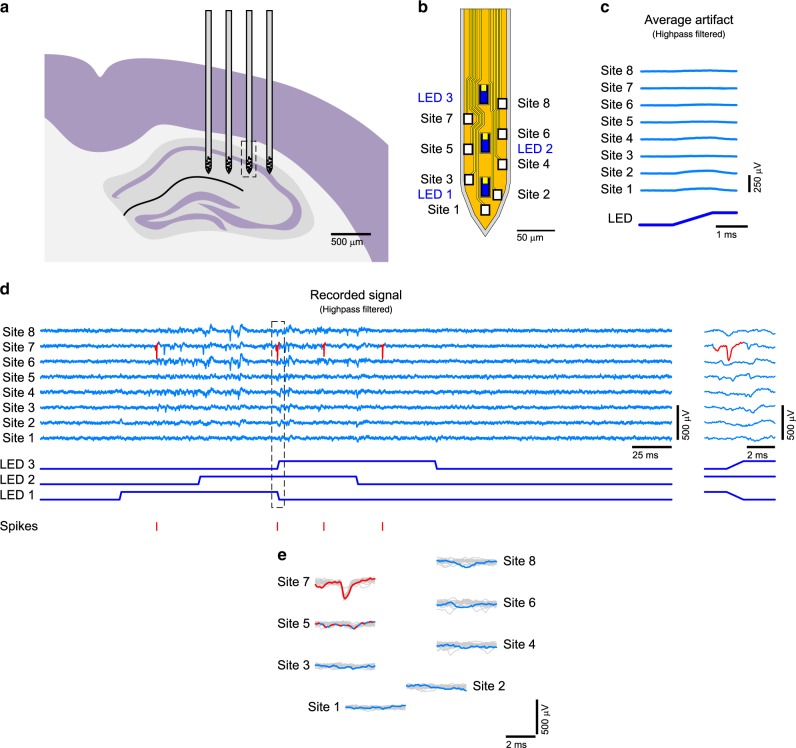
Stimulation-artifact-free in vivo opto-electrophysiology. **a** Schematic illustration of the location of implanted miniSTAR μLED optoelectrode inside the brain. The shank from which the data presented in parts **c**–**h** were collected is highlighted with a rectangle with dashed lines. **b** Schematic illustration of the tip of the miniSTAR optoelectrode. **c** Mean waveforms of stimulation artifact recorded on miniSTAR optoelectrodes, from channels that correspond to the electrodes on different locations on the tips. Shaded regions show one standard deviation away from the mean. LED drive signal with resulting LED surface irradiance of 3 mW mm^–2^ at the surface of the LED (radiant flux of 460 nW) was used. One LED was turned on and off at a time. **d** Traces of the recorded signals and the raster plots of sorted spikes. No signal processing, except for highpass filtering, was applied to the recorded signal. The inset on the right shows the magnified view of the region inside the rectangle with black dashed lines. **e** Magnified waveforms of the spikes recorded during the period highlighted in part **d**, overlaid on top of waveforms of 20 other spikes from the same neurons. Both spontaneous and light-induced spike waveforms are used without discrimination.

Following in vitro characterization, we demonstrated the successful elimination of supra-threshold stimulation artifact in vivo. We implanted a miniSTAR μLED optoelectrode in the brain of a mouse and positioned its tips in the CA1 region of the hippocampus (Fig. 6a). Once spontaneous spikes and the characteristic high-frequency oscillations (ripples) were detected from the recording electrodes on a shank, each LED on the shank was turned on with varying powers to identify the optimal intensity of optical stimulation to alter the spiking activity of neurons (“localized effect”) without inducing high-frequency oscillations due to synchronized firing of neuron populations^11^. Considering the typical duration of an action potential (<2 ms), we used a rise/fall time of 1 ms to ensure maximum reduction of stimulation artifact without loss of the temporal resolution in optical stimulation.

Stimulation with a 460-nW radiant flux (irradiance of 3 mW mm^–2^ at the surface of each µLED) induced strong light-induced responses in adjacent neurons. Optical stimulation with higher intensities induced high-magnitude (>100 μVpp) population bursting of multiple cells^11^, preventing identification of single neurons and analysis of the stimulation artifact. The mean waveform of the signal recorded from each channel corresponding to electrodes at different locations on the shank (Fig. 6b) during the onset of the 3-mW mm^–2^ optical stimulation is shown in Fig. 6c. No supra-threshold (>50 μVpp) stimulation artifact was observed from most channels.

After the characterization of the stimulation artifact resulting from the operation of individual LEDs, all three LEDs on the same shank were turned on and off in a sequence resulting in interleaved toggling of multiple LEDs (shown at the bottom of Fig. 6d). As shown in Fig. 6d, the series of optical pulses did not generate noticeable stimulation artifacts that would prevent either online detection of spikes or their offline spike sorting. Among a few potential neurons we identified with offline spike sorting^37^, we identified a putative pyramidal neuron (Supplementary Fig. 8), which fired within a short duration during which two LEDs were being toggled (in Fig. 6d, inside the rectangle with the dashed lines and in the inset on the right). No noticeable distortion of the spike waveform due to optical stimulation was observed (Fig. 6e).

## Discussion

The results of this study demonstrated the capability of high-spatiotemporal-resolution in vivo opto-electrophysiology with miniSTAR μLED optoelectrodes. We validated that the implementation of multi-metal-layer structure, the high-density boron doping of silicon substrate, and the transient pulse shaping of stimulation waveform can effectively suppress the stimulation artifact. Table 1 shows the summary of the characteristics of the components of the stimulation artifact and the results of our artifact suppression schemes.

**Table 1:** Characteristics of different components of the stimulation artifact on μLED optoelectrodes.

Type	Source	Magnitude	Shape (polarity)	Location dependence	Suppression scheme
EMI	n-GaN	Several mVs (combined with EMI from LED interconnects)	^-shaped (positive, or concave down)	No	Shielding layer
EMI	LED interconnects	Several mVs (combined with EMI from n-GaN)	^-shaped (positive, or concave down)	Yes	Shielding layer and transient pulse shaping
EMI	LED	Up to several 10^2 ^μVs	^-shaped (positive, or concave down)	Yes	Transient pulse shaping
PV	Silicon substrate	Up to several 10^2^ μVs	v-shaped (negative, or concave up)	No	Substrate doping

The magnitude of each stimulation artifact component was derived from the calculated difference in the magnitudes of the stimulation artifact measured from devices that did and did not utilize the suppression scheme indicated. The measurement of the stimulation artifact magnitude was made with μLED irradiance of 75 mW mm^–2^. The shape of the stimulation artifact indicates the shape observed in the recorded signal at the onset of the optical stimulation.

A few non-ideal features in the fabricated miniSTAR μLED optoelectrodes prevented the magnitude of the stimulation artifact from being further reduced. One imperfection is the existence of optical windows on the shielding layer, which allow EMI generated from LEDs to reach their neighboring recording electrode interconnects. The other non-ideal factor is that the shielding layer has non-zero resistance. The shielding layer, especially near the tips of the shanks, is not strictly an ideal ground due to a resistive voltage drop through the thin-film metal layer it is made of. This resistive voltage drop would make the voltage of the shielding layer fluctuate as the voltage of LED interconnect changes, and the shielding layer itself could have acted as a source of EMI. We observed that the magnitude of the EMI-induced stimulation artifact depends on not only the distance between the optical windows on the shielding layer and the electrode interconnect but also the distance between the LED interconnect layer and the electrode interconnect (Supplementary Fig. 9). This dependence suggests that a portion of the shielding layer on top of the LED interconnect layer might not be an ideal ground but instead might work as another EMI source. These non-idealities resulted in a less efficient reduction of EMI-induced artifact than that FEM electrostatic simulation predicted.

Residual EMI artifact might be able to be further suppressed with a few additional techniques. First, techniques to reduce the mutual capacitance between the recording electrode interconnect and the LED anode interconnect can be utilized. Increasing the distance between the recording electrode interconnects and the optical windows on the shielding layer (Fig. 4d) results in a reduction of the mutual capacitance. Alternatively, a pair of ground-connected traces serving as shielding guards could have been placed between the optical windows and the recording electrode interconnects. However, these measures to reduce the mutual capacitance would inevitably increase the width of the shanks. The shank width is the main limiting factor for high-density scaling of the device, which is required for larger-scale recording applications. Therefore, these options might not be considered optimal.

Methods to reduce electrode impedance might also be utilized for further reduction of the EMI-induced artifact. The current carrying the capacitively coupled signal is divided between two branches in the signal recording circuit each of which is terminated with the amplifier load and the electrode (Supplementary Fig. 10a). Therefore, lowering the electrode impedance would result in less current flowing through the amplifier load and thus reduction in the magnitude of the recorded voltage. The reduction of the electrode impedance with platinum-iridium (Pt-Ir) nanoparticle electroplating^38^ resulted in the reduction of the stimulation artifact magnitude measured in vitro (Supplementary Fig. 10b). Unfortunately, similar to many other site-level electrode surface modification techniques utilizing electrodeposition of conductive nanoparticles (e.g., Pt nanoparticle^39,40^ and PEDOT:PSS^41–43^), long-term in vivo stability of the technique has not yet been validated. Moreover, the low throughput of the site-by-site surface modification technique prevents the mass fabrication of the optoelectrode with low-impedance electrodes. Once a reliable, wafer-level electrode surface modification technique is developed, future versions of miniSTAR μLED optoelectrodes might be fabricated utilizing such technique and exhibit even smaller stimulation artifact.

Transient stimulation pulse shaping complements the two engineering schemes of structural changes implemented in the miniSTAR μLED optoelectrodes and effectively eliminates the stimulation artifact. With nearly zero amplitude artifact, miniSTAR optoelectrodes can be readily utilized for applications that require real-time event detection and closed-loop perturbation of neural circuits^1,21^. Still, the tradeoff between the temporal precision of optical stimuli and the amount of reduction in stimulation artifact should be taken into consideration. Most widely used opsins, including ChR2 and its early variants^44^, have slow kinetics and will not provide sub-millisecond-precision responses regardless of the precision of optical stimulus. Short-pulse optical stimulation protocols, similar to electrical protocols utilizing a train of sub-millisecond pulses^45^, might be found useful when used in combination with the recently developed fast-responding opsins^46,47^. Generation of such short (<2 ms) optical pulses may result in discernable signatures (>50 μVpp) in the recordings. These potential supra-threshold-amplitude artifacts, however, can easily be subtracted^48^ from the recorded trace since they occur at predetermined times and display identical waveforms. Therefore, residual stimulation artifact would not significantly compromise the quality of the recording.

In some applications, current-based LED driving might be more desirable than voltage-based driving. When driven with current pulses, the voltage change across the two terminals of an LED would follow the *I*–*V* characteristics of the LEDs. Therefore, current driving allows setting the non-zero off-state voltage across an LED, typically just below the LED turn-on voltage (≈ 2.8 V). We validated that the effect of the current-based driving of LEDs is similar to that with voltage-based driving of LEDs with non-zero low-level anode voltage (Supplementary Fig. 11). We further tested pulses with three different rise- and fall-time shapes: trapezoidal, sinusoidal, and sigmoidal. The shape of current pulses during on- to off-state transition did not significantly affect the magnitude of stimulation artifact. This result suggests that, if a stimulation pulse has a sufficiently low slew rate, the smoothing of pulse edges does not necessarily provide an additional reduction in the stimulation artifact magnitude.

Overall, our work demonstrates that stimulation artifact can be successfully suppressed using miniSTAR μLED optoelectrodes. This new device will allow performing high-temporal resolution in vivo opto-electrophysiology for the in-depth understanding of the interactions among the multiple components of neuronal circuits.

## Methods

### Shielded μLED optoelectrode fabrication and device assembly

Four-inch-diameter silicon wafers with different substrate boron doping densities (*N*_A_ ≈ 4 × 10^12^, 5 × 10^16^, and 1 × 10^20^ cm^–3^, respectively) with GaN/InGaN multi-quantum-well (MQW) LED layers epitaxially grown with metal-organic chemical vapor deposition (MOCVD) on top were purchased from Enkris Semiconductor (Suzhou, China). LED structures, including LED mesas, p- and n-GaN contacts and metallic interconnects, were first formed on the wafer. Repeated deposition of passivating dielectric layers and deposition of patterned metal layers formed additional metal layers. Consecutively, the top metal layer was passivated, and neural signal recording electrodes were defined. Finally, fabricated μLED optoelectrodes were thinned and released from the silicon wafer by double-sided plasma dicing process. The detailed fabrication process, including the list of tools used for each fabrication step, is provided in Supplementary Methods.

In order to prevent unwanted capacitive voltage coupling at the assembly level, we used four-layer printed circuit boards (PCBs) on which the traces for recorded neuronal signals and LED drive signals are separated by two ground-connected internal layers. The optoelectrodes were mounted on the PCBs and were electrically connected to the PCBs by wirebonding contact pads on the backend of the optoelectrode to the gold pads on the PCBs. After wirebonding, exposed wires were potted with epoxy (EPO-TEK 353ND and 353NDT, Epoxy Technology, Billerica, MA), and connectors (Omnetics Connector Corp., Minneapolis, MN), as well as the ground and the reference wires, were soldered to the PCBs to finalize the assembly process.

### MicroLED and recording electrode characterization

The electrical and optical properties of each μLED on the μLED optoelectrodes were characterized before in vitro and in vivo characterizations of the stimulation artifact. Both the current–voltage (*I* vs.*V*) and the irradiance-voltage (*E*_e_ vs. *V*) characteristics were measured for each μLED. First, an optical measurement system consisting of an integrating sphere (FOIS-1, Ocean Optics, Largo, FL) and a spectrometer (Flame, Ocean Optics) was built. A source meter (Keithley 2400, Keithley Instruments, Cleveland, OH) was then connected across the anode and the cathode pins of a μLED on the connector. The tips of the optoelectrode were lowered until the shanks were completely inside the integrating sphere, ensuring that all the light generated from the μLED can be collected. The DC voltage across the LED anode and the cathode terminals was swept from 0 V to 4 V, and the resulting current and the spectral flux of the μLED were measured. The radiant flux was calculated by integrating the spectral flux over wavelengths from 350 nm to 600 nm, and the irradiance on the surface of the μLED was then calculated by dividing the radiant flux by the μLED’s surface area (150 μm^2^).

The impedance (both the magnitude and the phase at 1 kHz) of each recording electrode on the μLED optoelectrode was measured using an Intan neural signal recording amplifier (RHD2132, Intan Technologies, Los Angeles, CA, on an Intan RHD2132 miniature neural signal amplifier headstage PCB) in 1× phosphate-buffered saline (PBS) solution (prepared using 10× PBS purchased from MP Biomedicals, Solon, OH). First, a small amount of PBS (~100 mL of 1× PBS solution) was poured into a small clear polystyrene container (530C-CRY, AMAC Plastic Products, Petaluma, CA). The μLED optoelectrode was lowered into the container until the bottom halves of the shanks (~2.5 mm) were submerged in the PBS. Exposed stainless steel tips at the loose ends of the ground and the reference wires were also submerged in the PBS. A neuronal signal recording system (RHD2000, Intan Technologies, with RHD2000 interface software v 1.5.2) conducted electrode impedance measurements.

### In vitro LED-induced artifact characterization

In vitro characterization was conducted inside room temperature 1× PBS solution in a clear acrylic (AMAC 530C-CRY) container. A μLED optoelectrode was lowered into the container until the bottom halves of the shanks were submerged in the PBS. The exposed stainless steel tips at the loose ends of the ground and the reference wires were also submerged in the PBS.

An Intan RHD2000 neuronal signal recording system with Intan RHD2000 interface software, in combination with an Intan RHD2132 headstage PCB, recorded stimulation artifacts at a 20-kHz sampling rate, while a function generator (33220 A, Keysight Technologies, Santa Rosa, CA) provided voltage pulses for LED driving. Fifty-millisecond long (5 Hz frequency, resulting in 25% duty ratio) rectangular voltage pulses were used as LED drive signals. The off-time (low-level) voltage, the on-time (high-level) voltage, the pulse rise time, and the pulse fall time were varied for different experiments. The experimental conditions used for each type of experiment are summarized in Supplementary Table 1. Before the LED drive signal was provided, the impedance (both the magnitude and the phase at 1 kHz) of each recording electrode on the μLED optoelectrode was measured using the Intan amplifier.

For the characterization of the effect of the shielding layer on the magnitude of in vitro LED-drive-induced artifact, we used μLED optoelectrodes with and without the shielding layer. Two one-metal-layer μLED optoelectrodes and two shielded μLED optoelectrodes, all of which were fabricated using the LED wafer with p^−^ silicon substrate (boron doped, *N*_A_ ≈ 5 × 10^16^ cm^–3^), were used. First, the high-level voltages required for the generation of a radiant flux of 230–11.5 μW (LED surface irradiance of 1.5–75 mW mm^–2^) were calculated. The high-level voltage of the LED drive pulse signal was varied according to the target irradiance, while the low-level voltage was fixed at 0 V and the rise time (as well as the fall time) was fixed as 5 ns (10–90%, equivalent to 6.25 ns of 0–100% rise and fall times).

For the characterization of the effect of the boron doping of the silicon substrate on the magnitude of in vitro LED-drive-induced artifact, we used shielded μLED optoelectrodes fabricated using LED wafers with different boron doping densities. Six shielded μLED optoelectrodes fabricated using LED wafers with FZ, p^−^, and p^+^ silicon substrate (two optoelectrodes from each wafer) were used. LED drive signals identical to those used for the characterization of the effect of the shielding layer were used.

For the characterization of the effect of the transient stimulation pulse shaping on the magnitude of in vitro LED-drive-induced artifact, we used miniSTAR μLED optoelectrodes, i.e., shielded μLED optoelectrodes fabricated using LED wafers with p^+^ silicon substrate. Two miniSTAR μLED optoelectrodes were used. The low-level anode voltage and the rise time of the LED drive pulse signal were varied, while the high-level anode voltage was fixed as 3.5 V. Low-level voltages of 0 V and 2.8 V were used, and rise and fall times (10–90%) between 5 ns and 1 ms were used.

For each experimental condition for each μLED, signals from the input channels of the neural signal amplifier IC were recorded for 30 s, so that artifact signals from more than 100 pulses can be recorded. Average artifact signal was calculated by first highpass filtering the signal to remove low-frequency fluctuations (with filters with 10 Hz and 250 Hz cutoff frequencies for wideband and highpass filtered signals, respectively) and calculating the average of the fifty 200-ms long segments in the middle of the 30 s period after the first 5 s of the recorded signal. Transient artifact magnitude was calculated from the difference between the maximum and the minimum values of the highpass filtered signal during the first 5-ms period from the point when the voltage changed from the off-level voltage. The mean transient artifact magnitude was calculated by taking the mean of the values from electrode whose impedance magnitudes are between 500 and 2 MΩ and the phases are between –80 ° and –55 ° at 1 kHz. Two μLED optoelectrodes from each cohort were used, and at least 21 electrodes per optoelectrode (out of 32 total, 25.83 on average) contributed to the calculation of the mean artifact magnitude. The mean 1 kHz magnitude and phase of the electrode impedance of the electrodes that contributed to the calculation of the mean artifact magnitude were 1.09 ± 0.09 MΩ and –68.2 ± 4.9 ° (mean ± SD, measured at 1 kHz).

### In vivo opto-electrophysiology

The animal procedures were approved by the Institutional Animal Care and Use Committee of the University of Michigan (protocol number PRO-7275). One male C57BL/6J mouse (32 g) was used for the in vivo characterization. The mouse was kept on a regular 12 h–12 h light–dark cycle and housed in pairs before surgery. No prior experimentation had been performed on this animal. Atropine (0.05 mg kg^–1^, s.c.) was administered after isoflurane anesthesia induction to reduce saliva production. The body temperature was monitored and kept constant at 36–37 °C with a DC temperature controller (TCAT-LV; Physitemp, Clifton, NJ, USA). Stages of anesthesia were maintained by confirming the lack of a nociceptive reflex. The skin of the head was shaved and the surface of the skull was cleaned by hydrogen peroxide (2%). A 1-mm diameter craniotomy was drilled at 1.5 mm posterior from bregma and 2 mm lateral of the midline. The dura was removed over the dorsal CA1 region of the hippocampus and the mouse was injected with AAV5, CaMKII-promoter-driven ChR2 (AAV5-CaMKIIa-hChR2(H134R)-EYFP), resulting in expression of ChR2 in pyramidal neurons. Viruses were purchased from the University of North Carolina Vector Core (UNC-REF). After the surgery, the craniotomy was sealed with Kwik-Sil (World Precision Instruments, Sarasota, FL) until the day of recording.

On the day of recording, the mouse was anesthetized with isoflurane, the craniotomy was cleaned, and a shielded μLED optoelectrode with p^+^ silicon substrate was lowered to the CA1 region of the hippocampus. Baseline recording was performed (30 min), after which simultaneous recording and stimulation were done using three μLEDs from one shank (as described in Results in more detail). 0.46 μW power, equivalent to 3 mW mm^–2^ irradiance at the surface of each μLED, was used to characterize the light-induced artifact in vivo and to alter the activity of neurons (more details are provided in Results). For the characterization of the stimulation artifact and the confirmation of the optical induction of neuronal activities, pulsed optical stimulation (100-ms long, 2 Hz, 100 pulses) was generated from each μLED. The (10–90%) rise and the fall times of each voltage pulse were set as 1 ms. After collecting sufficient data using optical stimulation from each μLED, a 500-ms long optical stimulation sequence involving switching on and off all the three μLEDs on the shank (whose details are provided in Results) were repeated 100 times. RHD2000 recording system with RHD2132 miniature neural signal amplifier headstage was used for the acquisition of data from all the recording electrodes (*n* = 32, 20 kS s^–1^ sampling rate). Keysight 33220A function generator provided voltage pulses for LED driving.

A custom MATLAB (MathWorks, USA) script was used to calculate the mean stimulation artifact. Wideband traces were first highpass filtered with a first-order filter with 250 Hz cutoff frequency to remove low-noise fluctuations. The average artifact signal from each recording channel was then obtained by averaging the middle 500-ms long segments (90 total segments out of 100).

The recorded data were then further analyzed for identification and clustering of action potentials. No manipulation in data (e.g. trimming of 1-ms long segments before and after the beginning and the ending of each pulsed optical stimulation) other than highpass filtering (at 500 Hz) of the baseband signal was conducted. Spikes were first detected and automatically sorted using the Kilosort algorithm^37^ and then manually curated using Phy to get well-isolated single units (multi-unit and noise clusters were discarded). To measure the effect of LED stimulation on neuronal activity, peristimulus time histograms (PSTHs) were built around stimulus onset (spike trains were binned into 10-ms bins). Baseline and light-induced firing rates were calculated for each single unit, in which the baseline was defined as light-free epochs (400 ms) between trials and the stimulation period as the light-on (100 ms). Wilcoxon signed-rank test was used to compare the mean firing rate per trial (*n* = 100 trials) during baseline and LED stimulation.

### Statistics and reproducibility

The details about the number of devices utilized for each in vitro experiment and the number of pulses from which the mean stimulation artifact waveform was calculated are provided in the sub-section titled In vitro LED artifact characterization. A summary of the number of the devices utilized and the experimental conditions utilized for each experiment is provided in Supplementary Table 1. Other information about the statistics, including the number of the electrodes from which the measurements were taken and thus the averages were calculated for each experiment, is provided in Supplementary Table 2. One device was utilized for the in vivo experiment.

### Reporting summary

Further information on research design is available in the Nature Research Reporting Summary linked to this article.

## Supplementary information

Supplementary Information

Reporting summary

## Data Availability

The datasets generated during and/or analyzed during the current study are available from the corresponding author on reasonable request.

## Code availability

All the custom scripts and model files that have been utilized for data analysis and device simulation are available from the corresponding author on reasonable request.
