# Supplementary material for: Artifact-free and high-temporal-resolution in vivo opto-electrophysiology with microLED optoelectrodes
Source: Nat Commun. 2020 Apr 28;11:2063. doi: 10.1038/s41467-020-15769-w (PMC7188816; doi:10.1038/s41467-020-15769-w)
Supplement: Supplementary file 2 — Reporting summary [file 41467_2020_15769_MOESM2_ESM.pdf]

nature research

Corresponding author(s): Euisik Yoon

Last updated by author(s): Mar 6, 2020

Reporting Summary

Nature Research wishes to improve the reproducibility of the work that we publish. This form provides structure for consistency and transparency in reporting. For further information on Nature Research policies, see Authors & Referees and the Editorial Policy Checklist.

Statistics

For all statistical analyses, confirm that the following items are present in the figure legend, table legend, main text, or Methods section.

n/a	Confirmed	
☐	☒	The exact sample size (*n*) for each experimental group/condition, given as a discrete number and unit of measurement
☐	☒	A statement on whether measurements were taken from distinct samples or whether the same sample was measured repeatedly
☐	☒	The statistical test(s) used AND whether they are one- or two-sided<br>*Only common tests should be described solely by name; describe more complex techniques in the Methods section.*
☐	☒	A description of all covariates tested
☐	☒	A description of any assumptions or corrections, such as tests of normality and adjustment for multiple comparisons
☐	☒	A full description of the statistical parameters including central tendency (e.g. means) or other basic estimates (e.g. regression coefficient) AND variation (e.g. standard deviation) or associated estimates of uncertainty (e.g. confidence intervals)
☐	☒	For null hypothesis testing, the test statistic (e.g. *F*, *t*, *r*) with confidence intervals, effect sizes, degrees of freedom and *P* value noted<br>*Give P values as exact values whenever suitable.*
☒	☐	For Bayesian analysis, information on the choice of priors and Markov chain Monte Carlo settings
☒	☐	For hierarchical and complex designs, identification of the appropriate level for tests and full reporting of outcomes
☐	☒	Estimates of effect sizes (e.g. Cohen's *d*, Pearson's *r*), indicating how they were calculated

Our web collection on statistics for biologists contains articles on many of the points above.

Software and code

Policy information about availability of computer code

Data collection	All electrical recordings were collected using Intan Technologies's (Los Angeles, CA, USA) RHD2000 interface software (v 1.5.2).
Data analysis	Raw data (.dat) were processed and analyzed using custom MATLAB (v R2018a, MathWorks, Natick, MA, USA.) scripts. All custom MATLAB scripts will be made available upon request to the corresponding author. Spike sorting was conducted in two steps, first using KiloSort (https://github.com/cortex-lab/KiloSort) and then with Phy (https://github.com/cortex-lab/phy).

For manuscripts utilizing custom algorithms or software that are central to the research but not yet described in published literature, software must be made available to editors/reviewers. We strongly encourage code deposition in a community repository (e.g. GitHub). See the Nature Research guidelines for submitting code & software for further information.

Data

Policy information about availability of data

All manuscripts must include a data availability statement. This statement should provide the following information, where applicable:

- Accession codes, unique identifiers, or web links for publicly available datasets
- A list of figures that have associated raw data
- A description of any restrictions on data availability

The datasets generated during and/or analyzed during the current study are available from the corresponding author on reasonable request.

Field-specific reporting

Please select the one below that is the best fit for your research. If you are not sure, read the appropriate sections before making your selection.

☒ Life sciences ☐ Behavioural & social sciences ☐ Ecological, evolutionary & environmental sciences

For a reference copy of the document with all sections, see nature.com/documents/nr-reporting-summary-flat.pdf

Life sciences study design

All studies must disclose on these points even when the disclosure is negative.

Sample size	Data obtained from one animal subject is presented in the in-vivo validation section of this manuscript. We hypothesized that there is no significant difference between in-vivo and in-vitro conditions that would contribute to discrepancy in the magnitude of measured stimulation artifact. The in-vivo validation experiment we carried out well replicated the result we observed in-vitro (negligible stimulation artifact). We are in process of preparing a separate paper from the results from an additional animal. The magnitude of the stimulation artifact measured from the additional animal was comparable to that from the first animal. The results will be made available in the separate paper upon its publication. Part of the data can be made available upon reasonable request in the form of partially processed data.
Data exclusions	We did not disclose data collected from another animal, whose result was similar to those presented in the in vivo validation section of this manuscript, due to a potential non-disclosure agreement for the separate paper we are preparing using the data collected in the experimental session.
Replication	The result of the additional in-vivo experiment we conducted well replicated the result of the data presented in this manuscript.
Randomization	No randomization was applied due to existence of only one experimental animal.
Blinding	The investigators were not blind to the group allocation during data collection and analysis due to existence of only one experimental animal.

Reporting for specific materials, systems and methods

We require information from authors about some types of materials, experimental systems and methods used in many studies. Here, indicate whether each material, system or method listed is relevant to your study. If you are not sure if a list item applies to your research, read the appropriate section before selecting a response.

Materials & experimental systems

n/a	Involved in the study	
☒	☐	Antibodies
☒	☐	Eukaryotic cell lines
☒	☐	Palaeontology
☐	☒	Animals and other organisms
☒	☐	Human research participants
☒	☐	Clinical data

Methods

n/a	Involved in the study	
☒	☐	ChIP-seq
☒	☐	Flow cytometry
☒	☐	MRI-based neuroimaging

Animals and other organisms

Policy information about studies involving animals; ARRIVE guidelines recommended for reporting animal research

Laboratory animals	One male C57BL/6J mouse (32 g) was used for in vivo characterization. The mouse was kept on a regular 12h - 12h light - dark cycle and housed in pairs before surgery. The animal was kept inside a mouse room at one of the University of Michigan Animal Housing units managed by Unit for Laboratory Animal Medicine (ULAM), and temperature and the relative humidity of the room were kept between 68 degrees and 76 degrees Fahrenheit and between 30 % and 70 %, respectively. AAV5, CaMKII promoter driven ChR2 (AAV5-CaMKIIa-hChR2(H134R)-EYFP) was injected prior to recording.
Wild animals	The study did not involve wild animals.
Field-collected samples	The study did not involve samples collected from the field.
Ethics oversight	The animal procedures were approved by the Institutional Animal Care and Use Committee of the University of Michigan (protocol number PRO-7275)

Note that full information on the approval of the study protocol must also be provided in the manuscript.
